# Inhibition of autophagy potentiates anticancer property of 20(S)-ginsenoside Rh2 by promoting mitochondria-dependent apoptosis in human acute lymphoblastic leukaemia cells

**DOI:** 10.18632/oncotarget.8285

**Published:** 2016-03-23

**Authors:** Ting Xia, Jiancheng Wang, Yingnan Wang, Yuanyuan Wang, Jianye Cai, Min Wang, Qidan Chen, Jia Song, Ziqi Yu, Wei Huang, Jianpei Fang

**Affiliations:** ^1^ Key Laboratory of Industrial Fermentation Microbiology, Ministry of Education, College of Biotechnology, Tianjin University of Science and Technology, Tianjin, P.R. China; ^2^ Center for Stem Cell Biology and Tissue Engineering, Key Laboratory for Stem Cells and Tissue Engineering, Ministry of Education, Sun Yat-Sen University, Guangzhou, P.R. China; ^3^ Department of Pediatrics, Sun Yat-sen Memorial Hospital, Sun Yat-Sen University, Guangzhou, P.R. China; ^4^ Key Laboratory of Malignant Tumor Gene Regulation and Target Therapy of Guangdong Higher Education Institutes, Sun Yat-Sen University, Guangzhou, P.R. China; ^5^ Department of Chemistry and Pharmacy, Zhuhai College, Jilin University, Zhuhai, P.R. China

**Keywords:** ginsenoside Rh2, acute lymphoblastic leukaemia, autophagy, apoptosis, mitochondria

## Abstract

Acute lymphoblastic leukaemia (ALL) is the most prevalent childhood malignancy. Although most children with ALL are cured, there is still a group of patients for which therapy fails owing to severe toxicities and drug resistance. Ginsenoside Rh2 (GRh2), a major bioactive component isolated from *Panax ginseng*, has been shown to have a therapeutic effect on some tumors. However, the molecular mechanisms of cell death induced by 20(S)-GRh2 in ALL cells remains unclear. In this study, we showed that 20(S)-GRh2 inhibited the cell growth and induced mitochondria-dependent apoptosis and autophagy. But it has no cytotoxic effect on human normal blood cells. Furthermore, autophagy plays a protective role in 20(S)-GRh2-induced apoptosis in ALL cell lines and human primary ALL cells. We demonstrated that either genetic or pharmacologic inhibition of autophagy could be more effective in reducing viability and enhancing 20(S)-GRh2-induced toxicity than 20(S)-GRh2 treatment alone. In addition, inhibition of autophagy could aggravate mitochondrial ROS generation and mitochondrial damage, and then accelerate mitochondria-dependent apoptosis. Taken together, these results suggest that inhibition of autophagy can sensitize ALL cells towards 20(S)-GRh2. The appropriate inhibition of autophagy could provide a powerful strategy to increase the potency of 20(S)-GRh2 as a novel anticancer agent for ALL therapy.

## INTRODUCTION

Acute lymphoblastic leukaemia (ALL) is the most common malignancy in childhood, accounting for 25% of all childhood cancers [1]. It can be regarded as clonal expansion of B- and T-lymphocytes arrested at an immature stage of differentiation a malignant disorder of lymphoid progenitor cells [2]. Precursor B-acute lymphoblastic leukaemia (pre B-ALL) is the most prevalent phenotype of ALL, and T-acute lymphoblastic leukaemia (T-ALL) associated with a poor outcome [3, 4]. However, current standard treatment consists of combination chemotherapy that is highly toxic to growing children, both in short and long terms [5–7]. To avoid serious side effects in chemotherapy and improve survival rates, novel anticancer agents are needed to address the emerging problem. Recent studies have shown that several natural traditional Chinese medicine (TCM) products can activate cell death pathway in cancer cells [8, 9].

Ginsenosides are extracted from the root of Panax ginseng C.A. Meyer, which are the major effective ingredients in ginseng [10, 11]. Based on the structural differences, ginsenosides can be classified 20(S)-protopanaxadiol (ginsenosides Rb1, Rb2, Rb3, Rc, Rd, Rh2, Rg3 and others) or 20(S)-protopanaxatriol (ginsenosides Re, Rg1, Rg2, Rh1 and others) compounds [12, 13]. Among these, GRh2 has been shown to have anticancer effects, and could inhibit cell growth and induce apoptosis in several cancer cell lines [13–16]. Furthermore, it has been reported that 20(S)-GRh2 exhibits stronger anticancer activity than 20(R)-GRh2 [17–18]. Due to its safety and no side-effects, GRh2 could be further developed as a useful drug for cancer therapy [19, 20]. Recently, researchers have found that 20(S)-GRh2 could induce autophagy when undergoing induced apoptosis in Hepatocellular carcinoma [21]. Our previous studies demonstrated that 20(S)-GRh2 could inhibit cell growth and induce apoptosis in pre B-ALL Reh cells [22]. In this report, we are interested to investigate whether autophagy was intervened in 20(S)-GRh2-induced apoptosis in human ALL cells.

Autophagy and apoptosis are catabolic pathways essential for organismal homeostasis, both of which play key roles in ultimate decisions of cancer cell fate [23]. Apoptosis, or type I PCD, involves the degradation of cellular constituents by a family of cysteine proteases called caspases [24]. As we all known, two core pathways exist to induce apoptosis, the extrinsic-death receptor pathway and intrinsic-mitochondrial pathway [25]. The mitochondrial pathway of apoptosis is seen as a common cause of tumor cell death induced by chemotherapeutic agents [26, 27]. The mitochondrial apoptotic pathway is characterized by reactive oxygen species (ROS) accumulation, loss of mitochondrial membrane potential (MMP), release of cytochrome c into the cytoplasm, which is followed by activation of caspase-9 and caspase-3 [28–30].

Similarly, autophagy is an evolutionarily conserved catabolic process of lysosomal degradation of cytoplasmic content in eukaryotes [31]. It is regulated by proteins including mammalian target of rapamycin (mTOR), phosphoinosmde-3-kinase (PI3K), GTPases, calcium and elements of protein synthesis machinery [32]. Recent studies have demonstrated that the existence of a complex relationship between autophagy and apoptosis, determining whether a cell will live or die in response to anticancer therapies [33, 34]. On one hand, autophagy can clear toxic elements and damage organelles, which suppresses apoptosis and prevents cell death [35]. On the other hand, it could also act as a facilitator of apoptosis via damaging mitochondria and contribute to cell death [36]. However, the molecular mechanisms of the interaction between autophagy and apoptosis induced by 20(S)-GRh2 remain unclear.

In the present study, we used autophagy inhibitor 3-methyladenine (3-MA), autophagy stimulator rapamycin (RAPA) and ATG5 knockdown to evaluate the potential role of autophagy in 20(S)-GRh2-induced apoptosis. We examined mitochondrial function and ROS generation to elucidate the interaction between autophagy and apoptosis induced by 20(S)-GRh2 in ALL. These findings would illuminate the anticancer property of 20(S)-GRh2 and provide a promising strategy for treatment of ALL.

## RESULTS

### Effect of 20(S)-GRh2 on cell proliferation in ALL cell lines and human normal blood cells

**Figure 1:**
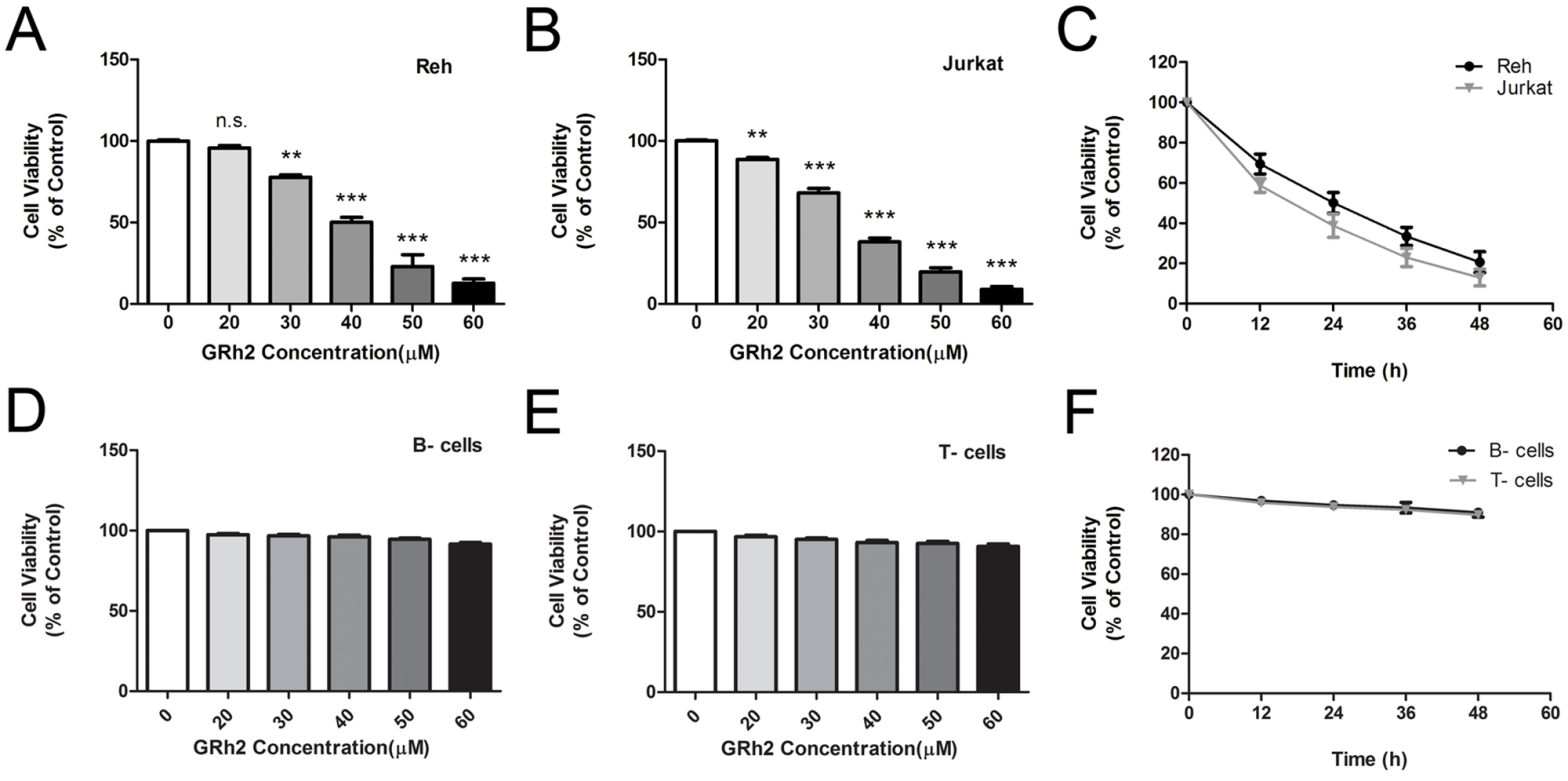
Effect of 20(S)-GRh2 on cell viability in ALL cell lines and human normal blood cells Reh **A.** and Jurkat **B.** cells were treated with various concentrations of 20(S)-GRh2 (0, 20, 30, 40, 50 and 60 μM) for 24 h. **C.** Reh and Jurkat cells were treated with 40 μM 20(S)-GRh2 for different times (12, 24, 36 and 48 h). The cell viability was assessed with cell counting kit-8 assay. Human normal B- cells **D.** and T- cells **E.** were treated with various concentrations of 20(S)-GRh2 for 24 h. **F.** Normal B- and T- cells were treated with 40 μM 20(S)-GRh2 for different times. Cell viability of every group was determined separately by cell counting kit-8 assay. Data are presented as mean ± SEM (n=3) for each group. ** p < 0.01, *** p < 0.001.

The effect of 20(S)-GRh2 on cell viability in ALL cell lines and human normal blood cells were investigated by cell counting kit-8 (CCK-8) assay. Cells were treated with increasing concentrations of 20(S)-GRh2 for 24 h. The results showed that 20(S)-GRh2 significantly reduced the viability of ALL cell lines in a dose-dependent manner (Figure 1A & 1B). The IC50 of 20(S)-GRh2 was about 40 μM for Reh cells and 35 μM for Jurkat cells. However, the viability of normal B- and T- cells were not obviously decreased (Figure 1D & 1E).

Then we chose 40 μM GRh2 to detect the cell growth at different time points. When cells were treated with 40 μM 20(S)-GRh2 for 48 h, the viability of Reh and Jurkat cells were decreased in a time-dependent manner (Figure 1C), whereas in normal B- and T- cells, the cell viability were not significantly decreased. (Figure 1F). Collectively, these results suggest that 20(S)-GRh2 inhibits cell proliferation of ALL cell lines and has no cytotoxic effect on human normal blood cells.

### 20(S)-GRh2 induces mitochondria-dependent apoptosis in ALL cells

**Figure 2:**
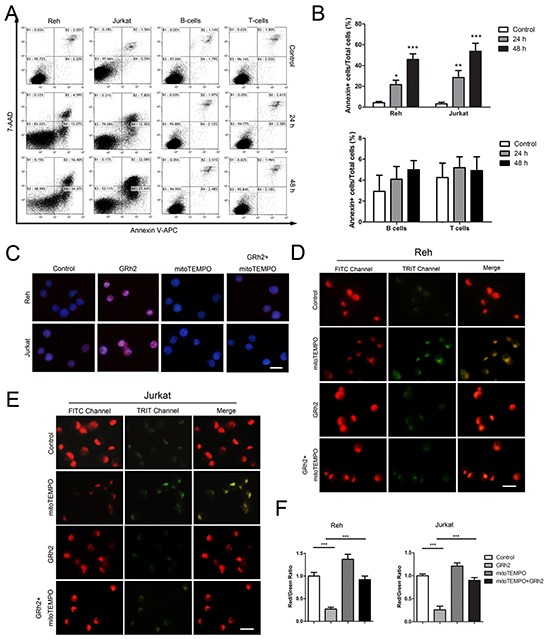
GRh2 induces mitochondria-dependent apoptosis in ALL cells **A.** Cells were treated with 40 μM 20(S)-GRh2 and harvested at the indicated time points. Representative dot plots of Annexin V/7-AAD staining are shown. Annexin V positive cells were analyzed by flow cytometer. **B.** The corresponding histograms were quantified by Image J. Data are reported as mean ± SEM (n=3) for each group. ** p<0.01, *** p<0.001: Bonferroni's post hoc test vs. control. **C.** Reh and Jurkat cells were treated with 40 μM 20(S)-GRh2 for 24 h in the presence or absence of 50 μM mitoTEMPO. Cells were stained with MitoSOX™ Red and Hoechst 33342. The fluorescence intensity was evaluated using fluorescent microscopy (Bar=10 μM). Reh **D.** and Jurkat **E.** cells were treated with different ligands as previously described and stained with JC-1. The fluorescence intensity was evaluated using fluorescent microscopy (Bar=10 μM). **F.** The ratio of Red/Green reflects MMP levels. The corresponding histograms were quantified by Image J. All data are represented as mean ± SEM (n=3) for each group.

First, we examined apoptotic cells induced by 20(S)-GRh2 via Annexin V and 7-AAD double staining. Cells were treated with 40 μM 20(S)-GRh2 for 48 h, and Annexin V positive cells were measured with flow cytometric analysis. The results showed that in Reh and Jurkat cells, the population of Annexin V positive cells was 16.66 ± 3.25 % and 20.70 ± 3.22 % at 24 h, respectively. After a 48 h treatment with 40 μM 20(S)-GRh2, the percentage of apoptotic cells was further increased to 50.87 ± 5.43 % at 24 h and 47.72 ± 5.02 %, respectively. However, in normal B- and T- cells, the apoptotic levels were not significantly increased after 24 h and 48 h 20(S)-GRh2 administration (Figure 2A & 2B). These data indicate that 20(S)-GRh2 can induce cell death in ALL cells but not in normal blood cells.

Then, in order to measure mitochondrial ROS levels triggered by 20(S)-GRh2 in ALL cells, we used MitoSOX™ Red reagent, which can detect superoxide inside mitochondria of live cells. The results showed that the red fluorescence intensity was obviously increased in 20(S)-GRh2-treated Reh and Jurkat cells, and then markedly attenuated when concurrent treatment with mitoTEMPO, a specific mitochondrial ROS inhibitor (Figure 2C). In addition, the levels of mitochondrial ROS were not obviously increased, when normal B- and T- cells were treated with 40 μM 20(S)-GRh2 for 24 h (Supplementary Figure S2A & S2B). On one hand, mitochondrial damage could lead to mitochondrial dysfunction. We used the sensitive fluorescent probe JC-1 as an indicator of mitochondrial dysfunction to examine the MMP levels. In fluorescence microscopic study, 20(S)-GRh2 induced a shift in the fluorescence spectrum of JC-1 from an aggregated form with orange-red fluorescence to a monomeric form with green fluorescence (Figure 2D & 2E), while mitoTEMPO rescued the reduction of red/green ratio in 20(S)-GRh2- treated Reh and Jurkat cells (Figure 2F). On the other hand, mitochondrial damage could trigger intrinsic apoptosis. We analyzed the expression of mitochondrial apoptosis-related proteins in 20(S)-GRh2-treated cells by western blot. We found that the expression levels of cytochrome c, cleaved caspase-9 and -3 proteins were upregulated; whereas the expression levels of these proteins were decreased by adding mitoTEMPO in 20(S)-GRh2-treated Reh cells (Supplementary Figure S1A & S1B). Therefore, these results suggest that 20(S)-GRh2 induces apoptosis through generation of mitochondrial ROS and mitochondrial signaling pathways in ALL cells.

### 20(S)-GRh2 induces autophagy in ALL cells

Firstly, we used monodansylcadaverine (MDC), a lysosomotropic compound to assess autophagy induction via the accumulation of MDC-labeled vacuoles. Reh cells were treated with increasing concentrations of 20(S)-GRh2 and stained with MDC, a fluorescent dye that selectively labels autophagic vacuoles (AVOs). As shown in Supplementary Figure S3A, in laser scanning confocal microscope study, MDC staining revealed that huge amount of AVOs formation occurred in Reh cells after 24 h 20(S)-GRh2 treatment. Consistent with this finding, in flow cytometric study, the percentage of MDC positive cells was also increased after 20(S)-GRh2 treatment (Supplementary Figure S3B & S3C).

**Figure 3:**
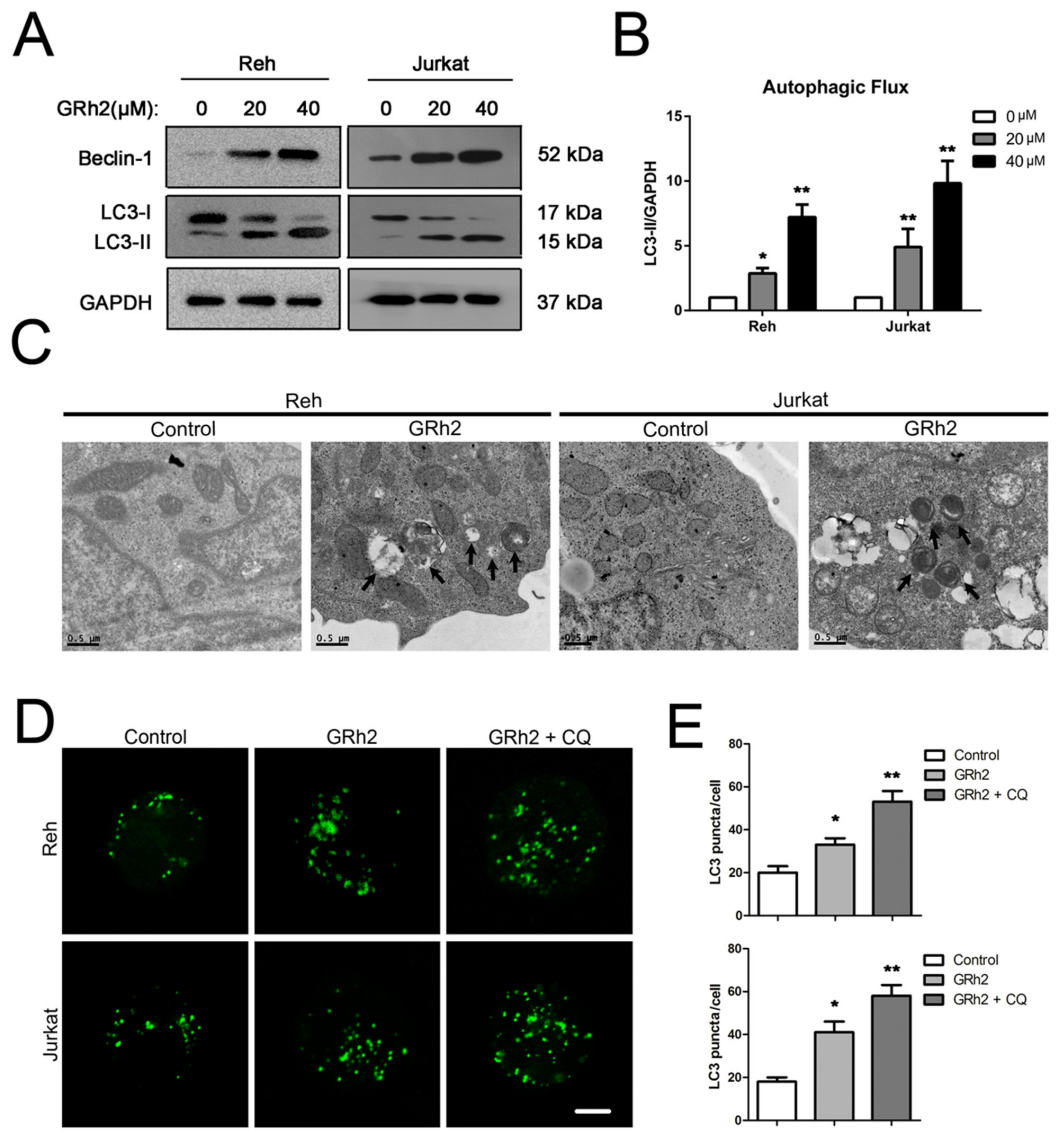
20(S)-GRh2 induces autophagy in ALL cells **A.** Reh and Jurkat cells were treated with different concentrations of 20(S)-GRh2 for 24h. The expression levels of LC3 were detected by western blot analysis. GAPDH was used as a loading control. **B.** Intensity of LC3-II and GAPDH levels was determined using densitometry analysis. Bar graphs shown represent normalized intensity levels of LC3-II (LC3-II/GAPDH) relative to no treatment control. Data are presented as mean ± SEM (n = 3) for each group. * p < 0.05, ** p < 0.01. **C.** Changes of ultrastructure in control and 40 μM 20(S)-GRh2 group were observed by transmission electron microscope (Bar=0.5 μM). Arrows indicate autophagosomes in 20(S)-GRh2-treated ALL cells. **D.** Cells were transfected with pEGFP-LC3 plasmids. After 24 h transfection, cells were treated with 40 μM 20(S)-GRh2 for 24 h in the presence or absence of 50 μM CQ. The fluorescence of GFP-LC3 was examined by laser scanning confocal microscope (Bar=5 μM). **E.** GFP-LC3 puncta in each cell was determined and bar graphs are representative of 100 cells. Data are presented as mean ± SEM (n = 3) for each group. * p < 0.05, ** p < 0.01.

To determine whether 20(S)-GRh2 treatment increased the autophagic flux in Reh and Jurkat cells, we conducted an analysis using autophagy protein LC3-II by western blot. As shown in Reh and Jurkat cells, the expression levels of LC3-II were increased at 24 h after treatment with increasing concentrations of 20(S)-GRh2 (Figure 3A & 3B). These data indicate that 20(S)-GRh2 treatment induces autophagic flux in ALL cells. Next, ultrastructural analysis by electron microscopy also revealed the formation of autophagosome in Reh and Jurkat cells at 24 h after 20(S)-GRh2 treatment (Figure 3C).

For further confirmation of autophagy, we performed an LC3 puncta assay by using pEGFP-LC3 and chloroquine (CQ), which inhibits late phase autophagy. The results showed that in Reh and Jurkat cells, 20(S)-GRh2 treatment significantly increased the number of GFP-LC3 puncta per cell compared with control, and CQ further enhanced the GFP-LC3 positive puncta upon 20(S)-GRh2 treatment (Figure 3D & 3E). Taken together, these results suggest that 20(S)-GRh2 can also induce autophagy in ALL cells.

### Autophagy is involved in 20(S)-GRh2-induced toxicity in ALL cells

We used 3-MA and RAPA to investigate the role of autophagy in 20(S)-GRh2-induced toxicity. 3-MA, an autophagic inhibitor, interferes with the formation of autophagosomes in mammalian cells via inhibition of the class III PI3K activity [37]. RAPA, an autophagic inducer, could inhibit mTOR, thus enhancing autophagy [38]. The involvement of autophagy was confirmed using LC3 puncta assay. As shown in Reh and Jurkat cells, the number of GFP-LC3 puncta per cell was significantly increased in cells treated with 3-MA and 20(S)-GRh2, while markedly decreased in cells treated with RAPA and 20(S)-GRh2, compared to 20(S)-GRh2 alone (Supplementary Figure S4A & S4B). In addition, the number of GFP-LC3 puncta per cell was not significantly increased after 24 h 20(S)-GRh2 administration, while markedly increased in normal B- and T-cells treated with RAPA compared with control (Supplementary Figure S2C & S2D).

**Figure 4:**
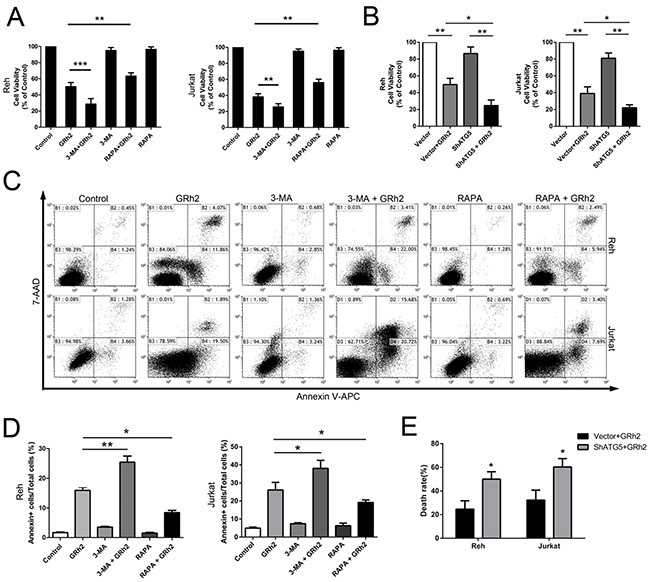
Autophagy is involved in 20(S)-GRh2-induced toxicity in ALL cells Cell viability was determined in Reh and Jurkat cells by the CCK-8 assay. **A.** Cells were treated with 40 μM 20(S)-GRh2 in the presence or absence of 3-MA or RAPA for 24 h. **B.** Cells transfected with vector or ShATG5 plasmid were exposed to 40 μM 20(S)-GRh2 for 24 h. Data are presented as mean ± SEM (n=3) for each group. * p < 0.05, ** p < 0.01, *** p < 0.001. **C.** The percentage of apoptotic cells was determined by FACS analysis. The flow cytometry dot plots showed the analysis of the percentage of apoptotic cells within ALL cell populations. **D.** The percentage of apoptotic cells in every group was analyzed and graphed. Data are presented as mean ± SEM (n=3) for each group. * p < 0.05, ** p < 0.01. **E.** Cells transfected with vector or ShATG5 plasmid were exposed to 40 μM 20(S)-GRh2 for 24 h. The percentage of dead cells with EthD-1 probe was determined by fluorescent microscopy. Images were analyzed using Image J. A minimum 500 cells were counted.

Then, combination of 3-MA and 20(S)-GRh2 was most effective in reducing cell viability compared with 20(S)-GRh2 alone, while induction of autophagy by RAPA significantly improved cell viability in the presence of 20(S)-GRh2 (Figure 4A). Moreover, combination of 3-MA and 20(S)-GRh2 effectively increased the percentage of apoptotic cells compared with 20(S)-GRh2 treatment alone, while induction of autophagy by RAPA dramatically attenuated apoptotic cell death (Figure 4C & 4D). These results indicate that inhibition of autophagy by 3-MA accelerates 20(S)-GRh2-induced toxicity to ALL cells, while induction of autophagy by RAPA protects ALL cells against 20(S)-GRh2-induced toxicity.

Next, to verify involvement of autophagy in 20(S)-GRh2-induced cytotoxicity, we specifically inhibited autophagy with shRNA targeting essential autophagy-related protein ATG5 in combination with 20(S)-GRh2. The results showed that shATG5 combined with 20(S)-GRh2 treatment markedly reduced the viability of Reh and Jurkat cells compared with cells treated with 20(S)-GRh2 and vector (Figure 4B). Furthermore, we used the LIVE/DEAD® viability/cytotoxicity assay to quantitate cell death. The results showed that combined treatment of autophagy inhibition with shATG5 and 20(S)-GRh2 significantly increased the percentage of dead cells compared with 20(S)-GRh2 and vector treatment (Figure 4E and Supplementary Figure S5A). These results suggest that in Reh and Jurkat cells, ATG5 knockdown can more effectively decrease cell viability and accelerate apoptotic cell death upon 20(S)-GRh2 treatment. Taken together, autophagy acts as an adaptive way to protect ALL cells against 20(S)-GRh2-induced cytotoxicity. Either genetic or pharmacologic inhibition of autophagy combined with 20(S)-GRh2 can be more effective in exacerbating 20(S)-GRh2-induced cytotoxicity than treatment with 20(S)-GRh2 alone.

### Inhibition of autophagy accelerates 20(S)-GRh2-induced apoptosis via mitochondrial ROS and mitochondrial damage

**Figure 5:**
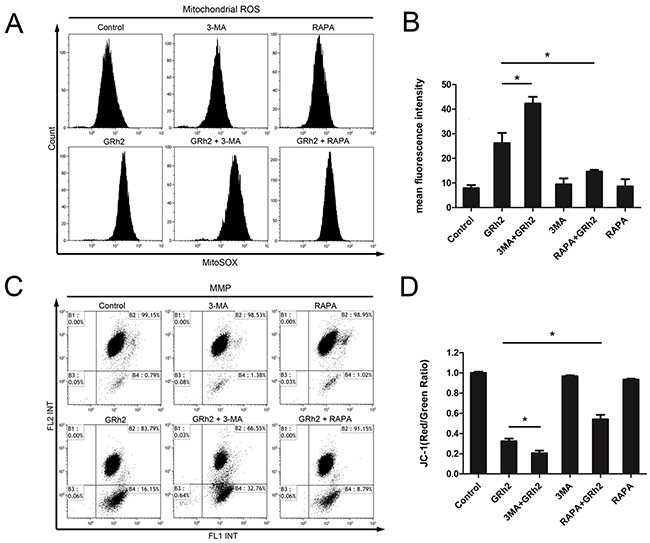
Inhibition of autophagy accelerates 20(S)-GRh2-induced apoptosis via mitochondrial ROS and mitochondrial damage Reh cells were treated with 40 μM 20(S)-GRh2 in the presence or absence of 3-MA or RAPA for 24 h. **A.** The MitoSOX™ Red fluorescence intensity was detected by flow cytometry. **B.** The corresponding histograms were quantified by Image J. All data are represented as mean ± SEM (n = 3) for each group. * p < 0.05. **C.** Representative pictures of JC-1 staining were shown, and the fluorescence intensity was detected by flow cytometry. **D.** The ratio of FL2/FL1 was reflects MMP levels. Data are represented as mean ± SEM (n=3) for each group. * p < 0.05.

To investigate the mechanisms of 20(S)-GRh2-induced toxicity to ALL cells, we measured mitochondrial ROS levels, MMP and mitochondrial-related apoptosis proteins. We found that in Reh cells, mitochondrial ROS generation was markedly increased in cells treated with 3-MA and 20(S)-GRh2, but significantly decreased in cells treated with RAPA and 20(S)-GRh2, compared to 20(S)-GRh2 alone (Figure 5A & 5B). Furthermore, combined treatment of 3-MA and 20(S)-GRh2 markedly accelerated depolarization of MMP, but combination of RAPA and 20(S)-GRh2 significantly attenuated it, compared with 20(S)-GRh2 treatment alone (Figure 5C & 5D). In addition, combined treatment of 3-MA and 20(S)-GRh2 dramatically increased the expression levels of mitochondria-associated pro-apoptotic proteins compared with 20(S)-GRh2 treatment alone, while combination of RAPA and 20(S)-GRh2 markedly decreased the expression levels of these proteins in Reh cells (Supplementary Figure S6A & S6B).

Next, we used shRNA to knockdown autophagy gene products ATG5 to confirm the role of mitochondrial ROS in 20(S)-GRh2-induced toxicity. The results showed that in Reh cells, shATG5 combined with 20(S)-GRh2 treatment markedly increased the fluorescence intensity compared with cells treated with 20(S)-GRh2 and control vector (Supplementary Figure S7A & S7B). In addition, combined treatment of autophagy inhibition with shATG5 and 20(S)-GRh2 significantly accelerated the loss of MMP compared with 20(S)-GRh2 and control vector treatment (Supplementary Figure S7C & S7D). These results taken together indicate that either genetic or pharmacologic inhibition of autophagy combined with 20(S)-GRh2 aggravates accumulation of mitochondrial ROS and mitochondrial damage, thus promoting apoptosis.

### Effect of autophagy on 20(S)-GRh2-induced apoptosis in primary ALL cells

**Figure 6:**
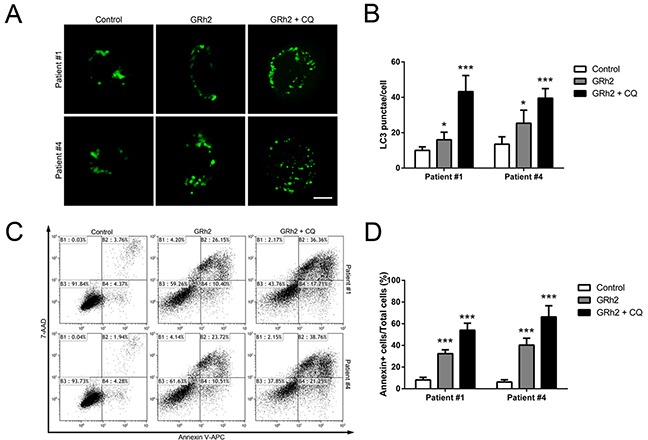
Autophagy plays a protective role in 20(S)-GRh2-induced apoptosis in human primary ALL cells Cells were transfected with pEGFP-LC3 plasmids. After 24 h transfection, cells were treated with 40 μM 20(S)-GRh2 in the presence or absence of CQ for 24 h. **A.** In primary B- ALL and T- ALL cells, the fluorescence of GFP-LC3 was examined by laser scanning confocal microscope (Bar=5 μM). **B.** GFP-LC3 puncta in each cell was determined and bar graphs are representative of 100 cells. Data are presented as mean ± SEM (n = 3) for each group. * p < 0.05, ** p < 0.01. **C.** The percentage of apoptotic cells was determined by FACS analysis. **D.** The percentage of Annexin V positive cells in every group was analyzed and graphed. Data are presented as mean ± SEM (n=3) for each group. * p < 0.05, ** p < 0.01.

We also evaluated the effect of autophagy on 20(S)-GRh2-induced apoptosis in primary ALL cells. Firstly, we investigated whether treatment with 20(S)-GRh2 could induce autophagy in primary ALL cells. As shown in Figure 6A & 6B, both primary B- ALL and T- ALL cells treated with 20(S)-GRh2 showed increased number of GFP-LC3 puncta per cell compared with untreated cells. CQ further enhanced GFP-positive puncta upon 20(S)-GRh2 treatment, indicating that 20(S)-GRh2 treatment induced autophagic flux. Next, we used CQ to determine the effect of autophagy on 20(S)-GRh2-induced apoptosis in primary ALL cells. Our data showed that 20(S)-GRh2 treatments alone significantly increased the percentage of apoptotic cells compared with control, while the combination of CQ and 20(S)-GRh2 further increased the apoptotic levels (Figure 6C & 6D). These results taken together indicate that inhibition of autophagy by CQ exacerbates 20(S)-GRh2-induced apoptosis in primary ALL cells.

## DISCUSSION

ALL is the most common childhood tumor, accounting for almost 30% of pediatric cancers [39]. As a step in overcoming the limitation in chemotherapy, innovative approaches are needed to further improve survival rates and to avoid serious side effects. Ginsenosides, the major pharmacologically active ingredients of ginseng, have been shown to have multiple medical effects such as anti-oxidative, anti-inflammation, anticancer and other health-improving activities [40, 41]. GRh2 is a pure compound extracted from ginsenosides and has anticancer activity [13–16, 42]. In this study, our objective is to explore the relationship between apoptosis and autophagy in 20(S)-GRh2-treated ALL cells, which could provide a novel anticancer agent for ALL therapies.

Recently, it has been reported widely that GRh2 could inhibit tumor cells proliferation and induce tumor cells apoptosis [43–45]. The results presented here suggested that in human ALL cells, 20(S)-GRh2 inhibited cell growth in a dose- and time-dependent manner, while had no cytotoxic ability to human normal blood cells. Both apoptosis and autophagy play essential roles in the development of organs, homeostasis, and cancer. A comprehensive understanding of autophagy and apoptosis is essential for the development of effective cancer therapeutics [46, 47]. Consistent with previous studies, our data were confirmed that 20(S)-GRh2 induced apoptosis through mitochondrial signaling pathway in ALL cells. Meanwhile, we reported a novel function of 20(S)-GRh2, induction of autophagy, as shown by cell morphological changes and increased autophagic flux in ALL cells. But 20(S)-GRh2 can not induce autophagy in normal blood cells. Therefore, we demonstrated that autophagy and apoptosis were both induced by 20(S)-GRh2 in ALL cells.

The intricate relationship between autophagy and apoptosis is complex and varies with cell and stress distinction [48, 49]. Some recent studies have focused on the intricate relationship between drug-induced autophagy and apoptosis. Several contradictory views of the effects of autophagy on chemotherapy-induced apoptosis in cancer have been reported [50, 51]. Chemotherapy-induced autophagy protects cells from apoptosis in some contexts [52], but promotes apoptosis in the others [53]. To explore the interplay between apoptosis and autophagy in 20(S)-GRh2-treated ALL cells, we used 3-MA, RAPA and ATG5 knockdown to manipulate autophagy. We demonstrated that inhibition of autophagy by 3-MA sensitizes ALL cells to 20(S)-GRh2, while induction of autophagy by RAPA protects cells against apoptosis. In addition, we found that ATG5 knockdown further enhanced cytotoxicity of 20(S)-GRh2 to ALL cells. Therefore, these findings suggest that autophagy plays a protective role in 20(S)-GRh2-induced apoptosis, and either genetic or pharmacologic inhibition of autophagy can effectively sensitize ALL cells to 20(S)-GRh2.

Then, to illuminate the molecular mechanisms of interaction between apoptosis and autophagy in 20(S)-GRh2-treated ALL cells, we further investigated mitochondrial ROS levels and mitochondrial dysfunction. ROS mainly generate inside mitochondria, whereas excess ROS generation could cause mitochondrial damage [54, 55]. Some links between apoptosis and autophagy are indicated via mitochondria [56]. It is reported that the elimination of damaged mitochondria by autophagy would prevent the release of proapoptotic substances from mitochondria, thus preventing apoptosis. In the absence of such clean up, the release of molecules like cytochrome c and apoptosis-inducing factor (AIF) from damaged mitochondria would lead to apoptosis [57, 58]. In this study, we found that inhibition of autophagy combined with 20(S)-GRh2 markedly increased mitochondrial ROS generation and accelerated dissipation of MMP in ALL cells, while induction of autophagy combined with 20(S)-GRh2 significantly alleviated 20(S)-GRh2-induced mitochondrial ROS generation and mitochondrial damage. In addition, mitochondrial damage further caused the release of cytochrome c and mitochondrial-related apoptosis proteins. Taken together, these results indicate that autophagy can help to clean up damaged mitochondria and play a protective role against 20(S)-GRh2-induced apoptosis. Inhibition of autophagy would aggravate mitochondrial damage, thus promoting mitochondria-dependent apoptosis in ALL cells.

Moreover, we extended our study to find out whether autophagy played a role in 20(S)-GRh2-induced apoptosis in primary ALL cells. We found that 20(S)-GRh2 treatment could increase autophagic flux in either primary B- ALL or T- ALL cells. Inhibition of autophagy by CQ sensitized those cancer cells towards 20(S)-GRh2. Therefore, our data suggest that inhibition of autophagy combined with 20(S)-GRh2 treatment would be more effective, and closer for future clinical application.

In view of the above arguments and the new data presented herein, we strongly propose that autophagy plays a protective role in 20(S)-GRh2-induced apoptosis in ALL cell lines and human primary ALL cells. Either genetic or pharmacologic inhibition of autophagy can be more effective in reducing viability and enhancing 20(S)-GRh2-induced toxicity to ALL cells. In addition, inhibition of autophagy could accelerate mitochondria-dependent apoptosis by stimulating mitochondrial ROS generation, promoting the loss of MMP and upregulation of mitochondria-dependent apoptosis in ALL cells. Collectively, this study suggests that inhibition of autophagy could potentiate anticancer property of 20(S)-GRh2 and provide a novel strategy for ALL therapy in future.

## MATERIALS AND METHODS

### Reagents and antibodies

20(S)-GRh2 (purity 99.48%) was purchased from Beijing North Carolina Chuanglian Biological Technology Research Istitute (Beijing, China). MitoTEMPO was obtained from Santa Cruz Biotechnology (Dallas, TX, USA), 3-MA, CQ and RAPA were purchased from Sigma.

The primary antibodies against rabbit cytochrome c, caspase-9, cleaved caspase-3, and the secondary horseradish peroxidase (HRP)-labeled goat-anti-mouse and goat-anti-rabbit antibodies, were purchased from Cell Signaling Technology (Danvers, MA, USA). Antibodies against rabbit cleaved caspase-9, GAPDH, β-actin, Tubulin were purchased from Bioworld Technology (NanJing, China). Antibodies against rabbit LC-3, Atg5 and beclin-1 were obtained from Abcam Company (Cambridge, MA, USA).

### Samples and cell culture

Bone marrow (BM) samples were derived from 6 newly diagnosed cases, untreated patients with B-ALL and T-ALL. Peripheral blood (PB) samples were obtained from 3 healthy individuals. All human BM and PB samples were obtained with written informed consent. Mononuclear cells isolated from BM and PB through density gradient centrifugation on standard Ficoll-HyPaque. T-cells and B-cells were sorted from peripheral blood mononuclear cells (PBMC) by FACS (BD Bioscience Influx, Franklin Lakes, NJ, USA). In brief, the PBMCs were incubated with FITC-conjugated anti-CD3, PE-conjugated anti-CD19 and purified by FACS to obtain the CD3^+^ T-cells and CD19^+^ B- cells, respectively. The purities of the sorted cells were >95%.

Human pre-B-ALL cell line Reh and T-ALL cell line Jacket were purchased from Cell Bank of Chinese Academic of Science (Shanghai, China). Cells were cultured at 37°C and 5% CO2 in RPMI 1640 medium (Hyclone, Logan, Utah, USA), supplemented with 10% fetal bovine serum (FBS, Gibco, Grand Island, NY, USA), Penicillin/Streptomycin (1:100, Sigma, St. Louis, MO, USA).

### Cell viability assays

Cell viability was determined by CCK-8 (Dojin Laboratories, Kumamoto, Japan) assay in different cells. Cells (5 × 10^5^ /mL) were plated on a 96-well (100 μL /well) microplate and treated with ligands for required time. Cells were added with 10 μL CCK-8 solution, and incubated for 4 h at 37°C. Absorbance in each well was quantified at 450 nm using an automated enzyme-linked immunosorbent assay (ELISA) reader (Tecan, Salzburg, Austria).

### The cytotoxicity assay

Cells transfected with vector or ShATG5 plasmid were exposed to 40 μM GRh2 for 24 h and stained using the LIVE/DEAD® viability/cytotoxicity assay kit (Invitrogen, Carlsbad, CA, USA) to analyze for the presence of live or dead cells. Briefly, calcein AM probe is conversed to the intensely fluorescent calcein in living cells, producing a green fluorescence. While, EthD-1 probe enters cells with damaged membranes, producing a red fluorescence by binding to nucleic acids. Cells were then stained with nuclear Hoechst 33342. The percentage of dead cells with EthD-1 probe was determined in high-power fields of each sample. Images were analyzed using Image J. A minimum 500 cells were counted.

### Annexin V/7-amino-actinomycin D (7-AAD) flow cytometry assay

Cells were treated with different ligands for required time. Cells were washed twice with cold PBS and resuspended in binding buffer 500 μL. Annexin V-APC and 7-Amino-actinomycin D (7-AAD, BD Pharmingen, San Diego, CA, USA) were added, away from light for 15 min at room temperature. The cells were immediately evaluated by flow cytometry (FACScan; Becton Dickinson, San Diego, CA, USA) within one hour. Annexin V+ and 7-AAD− cells were designated as early apoptotic cells and Annexin V+ and 7-AAD+ cells were designated as late apoptotic cells.

### Detection of autophagic vacuoles by MDC staining

A fluorescent compound, MDC (Sigma, St. Louis, MO, USA), has served as a useful fluorescent marker for autophagic vacuoles. Reh cells (5×10^5^/well) were cultured in 6-well culture plates and treated without or with 20(S)-GRh2 (20 and 40 μM) for 24 h, then incubated with 50 μM MDC in PBS at 37°C for 1 h. After washing with PBS, the stained cells were immediately observed under a laser scanning confocal microscope (Zeiss, Germany) and analyzed by flow cytometry.

### Detection of GFP-LC3

Human pEGFP-LC3 plasmid (Addgene plasmid 24920), which encodes a fusion protein of enhanced green fluorescent protein (EGFP) and LC3, was obtained from Addgene (Cambridge, MA, USA). Cells were seeded in 12-well plates and transfected with pEGFP-LC3 plasmid using Lipofectamine 2000 (Invitrogen) following the manufacturer's instructions. After 24 h transfection, cells were treated with different ligands for 24 h. The fluorescence of GFP-LC3 was examined by laser scanning confocal microscope. Autophagic flux was determined by evaluating the punctuated pattern of GFP (punctae/cell were counted).

### Knockdown of ATG5

ATG5 expression was knockdown by transfected with shRNA retroviruses. The shRNA and sequence was 5′-GCCATCAATCGGAAACTCATG-3′. Retrovirus particles were obtained in HEK293T cells according to the manufacturer's instructions. Cells were seeded into six-well plates and transfected with either empty or shRNA retroviruses using X-tremeGENE HP reagent (Roche Applied Science, Indianapolis, IN, USA), then stably transduced cells were selected by puromycin antibiotics in the medium.

### Measurement of mitochondrial ROS generation

The formation of mitochondrial ROS was measured using MitoSOX™ Red (Life technologies) assay. MitoSOX™ Red reagent is oxidized by superoxide once inside the mitochondria, and is converted to a fluorogenic oxidation product upon binding to nucleic acids [39]. For microscopy, cells were treated with 40 μM GRh2 for 24 h in the presence or absence of 50 μM mitoTEMPO. After staining with MitoSOX™ Red, cells were stained with Hoechst 33342. Microscopic images of fluorescence were collected using a fuorescence microscopy (IX-81; Olympus Corp., Tokyo, Japan). For flow cytometry, cells were treated with different ligands for 24 h. After staining, MitoSOX™ Red fluorescence was analyzed by flow cytometry.

### Detection of mitochondrial membrane potential

Cells were cultured in 12-well plates (5×10^5^/well) and treated with different ligands. In ALL cells, fluorescent probe JC-1 (Sigma, Louis, MO, USA) was used to estimate MMP. In cells treated with vector or shATG5, the MMP was determined using the fluorescent MitoTracker probe (Invitrogen). After 24 h incubation, cells were stained with JC-1 or MitoTracker in the dark at 37°C for 30 min and immediately observed under a fuorescence microscopy or analyzed by a FACScan flow cytometer.

### Transmission electron microscopy

After treatment with 40 μM GRh2 for 24 h, Reh and Jurkat cells, including control, were collected and fixed with 2.5% glutaraldehyde solution (pH7.4) for 2 h, post-fixed with 1% osmium tetroxide for 1 h, and then washed. After dehydration in a growing gradient of ethanol (30, 50, 70 and 95%, 5 min for each step), impregnation steps and inclusion were performed in Epon and finally polymerized at 60°C for 48 h. Ultrathin sections were stained with uranyl acetate and lead citrate for observation under a Tecnai G2 Spirit Twin transmission electron microscope (FEI, USA) at an accelerating voltage of 80 kV.

### Western blot analysis

After treatment, cells were collected and lysed for 30 min in RIPA buffer. The samples were heated to 100°C for 5 min and placed briefy on ice. A total of 20 μL of the supernatant was loaded onto a 12% sodium dodecyl sulfate-polyacrylamide gel electrophoresis (SDS-PAGE) gel. After electrophoresis, the protein was electrotransferred to a Hybond-ECL polyvi-nylidene fuoride membrane. The membrane was blocked with 5% milk for 1 h, and incubated overnight with primary antibodies, followed by horseradish peroxidase (HRP)-conjugated secondary antibodies for 1 h at room temperature. The membranes were incubated with HRP-conjugated secondary antibodies for 1 h at room temperature. The immunoreactive bands were detected with an enhanced chemiluminescence.

### Statistical analyses

All data were expressed as the means± the standard error of the mean (SEM). Statistical analyses were performed using Prism 5.03 (GraphPad Software Inc., San Diego, CA, U.S.A.). The data were examined using Student's t-test or one-way ANOVA followed by the Bonferroni *post hoc* test. A p value less than 0.05 was considered significant. The levels of significance were set at p < 0.05, p < 0.01 or p < 0.001.

## SUPPLEMENTARY FIGURES


