# Supplementary material for: Inhibition of autophagy potentiates anticancer property of 20(S)-ginsenoside Rh2 by promoting mitochondria-dependent apoptosis in human acute lymphoblastic leukaemia cells
Source: Oncotarget. 2016 Mar 23;7(19):27336–49. doi: 10.18632/oncotarget.8285 (PMC5053654; doi:10.18632/oncotarget.8285)
Supplement: Supplementary file 1 [file oncotarget-07-27336-s001.pdf]

SUPPLEMENTARY FIGURES

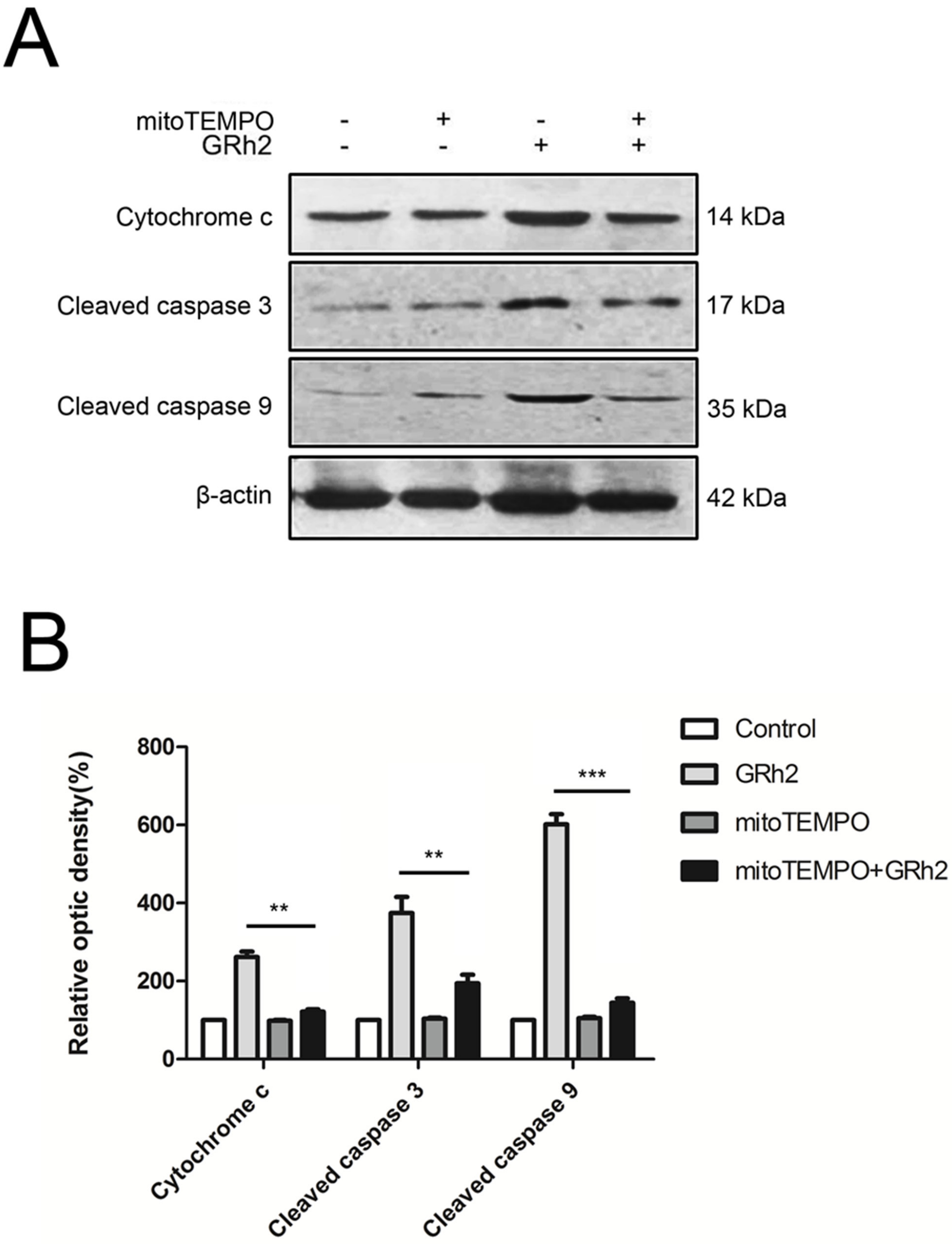

Supplementary Figure S1: Expression levels of mitochondrial apoptosis-related proteins in 20(S)-GRh2-treated Reh cells. A. Reh cells were treated with 40 μM 20(S)-GRh2 for 24 h in the presence or absence of 50 μM mitoTEMPO. The expression of cleavage of cytochrome c and caspase-9, -3 were detected by western blot analysis. Antibody against β-actin used as a loading control. **B.** The corresponding histograms were quantified by Image J. Data are reported as mean ± SEM (n=3) for each group. ** $p < 0.01$, *** $p < 0.001$.

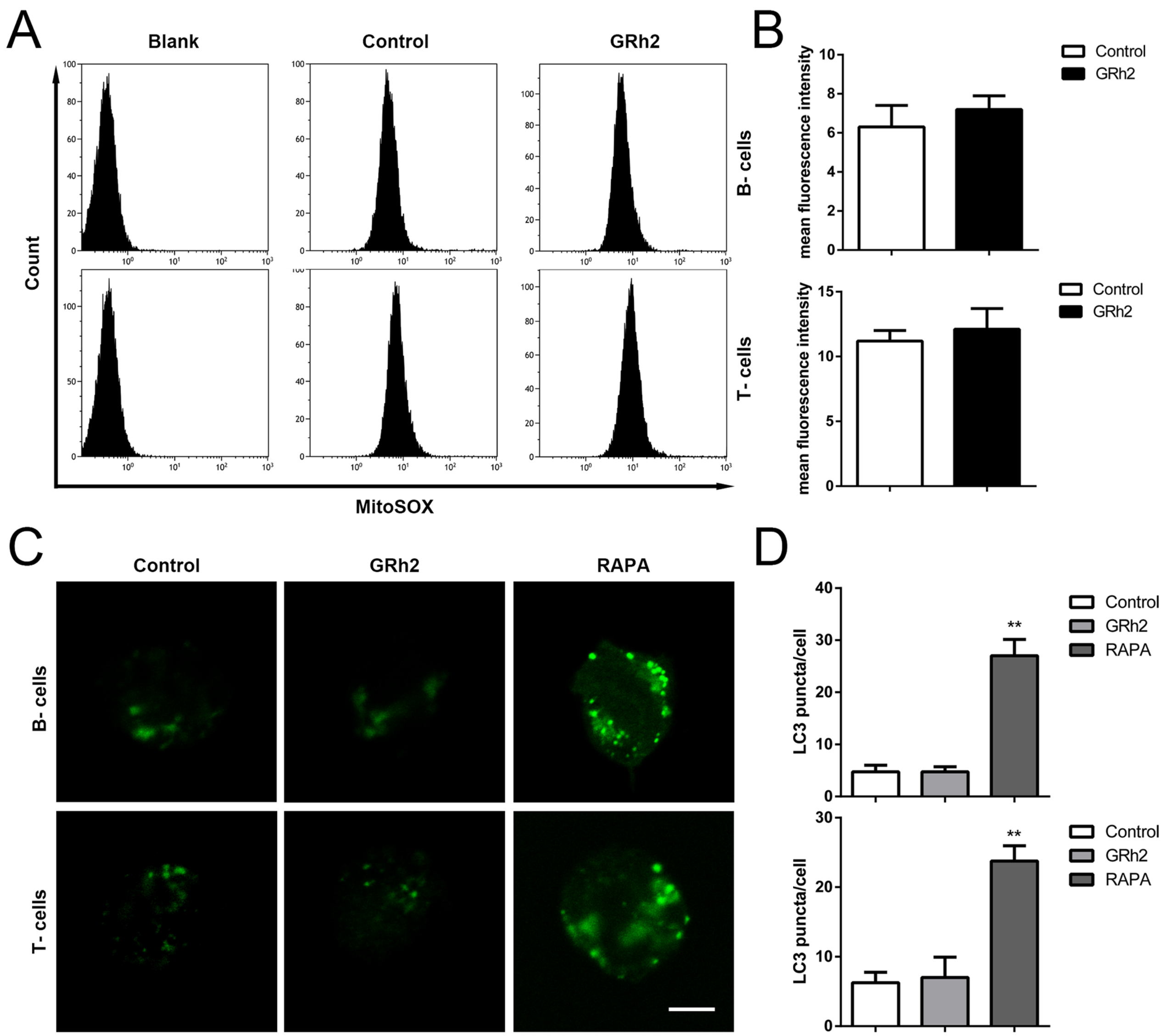

Supplementary Figure S2: The levels of mitochondrial ROS and autophagy in human normal blood cells after treated with 20(S)-GRh2. Normal B- and T- cells were treated with 40 μM 20(S)-GRh2 for 24 h. **A.** The MitoSOX™ Red fluorescence intensity was detected by flow cytometry. **B.** The corresponding histograms were quantified by Image J. All data are represented as mean ± SEM (n = 3) for each group. **C.** The fluorescence of GFP-LC3 was examined by laser scanning confocal microscope (Bar=5 μM). **D.** GFP-LC3 puncta in each cell was determined and bar graphs are representative of 100 cells. Data are presented as mean ± SEM (n = 3) for each group. ** $p < 0.01$.

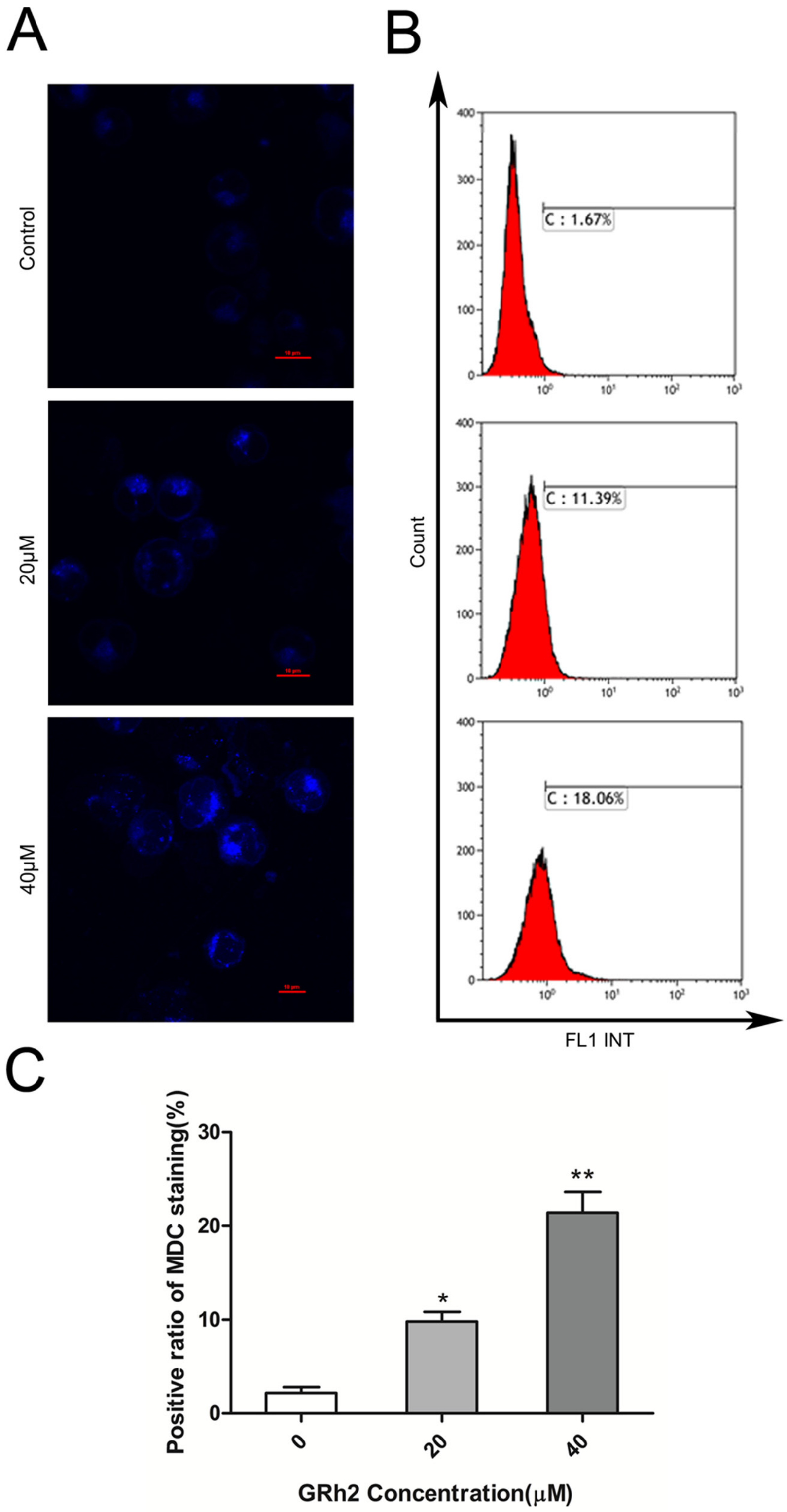

Supplementary Figure S3: 20(S)-GRh2 induces the accumulation of autophagic vacuoles in Reh cells. Reh cells were treated with different concentrations of 20(S)-GRh2 for 24h, and stained with MDC. **A.** The changes in cellular autophagic vacuole of every group were observed by laser scanning confocal microscope (Bar=10 µM). **B.** The ratio of MDC-positive cells was measured by flow cytometry in 20(S)-GRh2-treated Reh cells. **C.** The corresponding histograms were quantified by Image J. All data are represented as mean ± SEM ($n = 3$) for each group. * $p < 0.05$, ** $p < 0.01$.

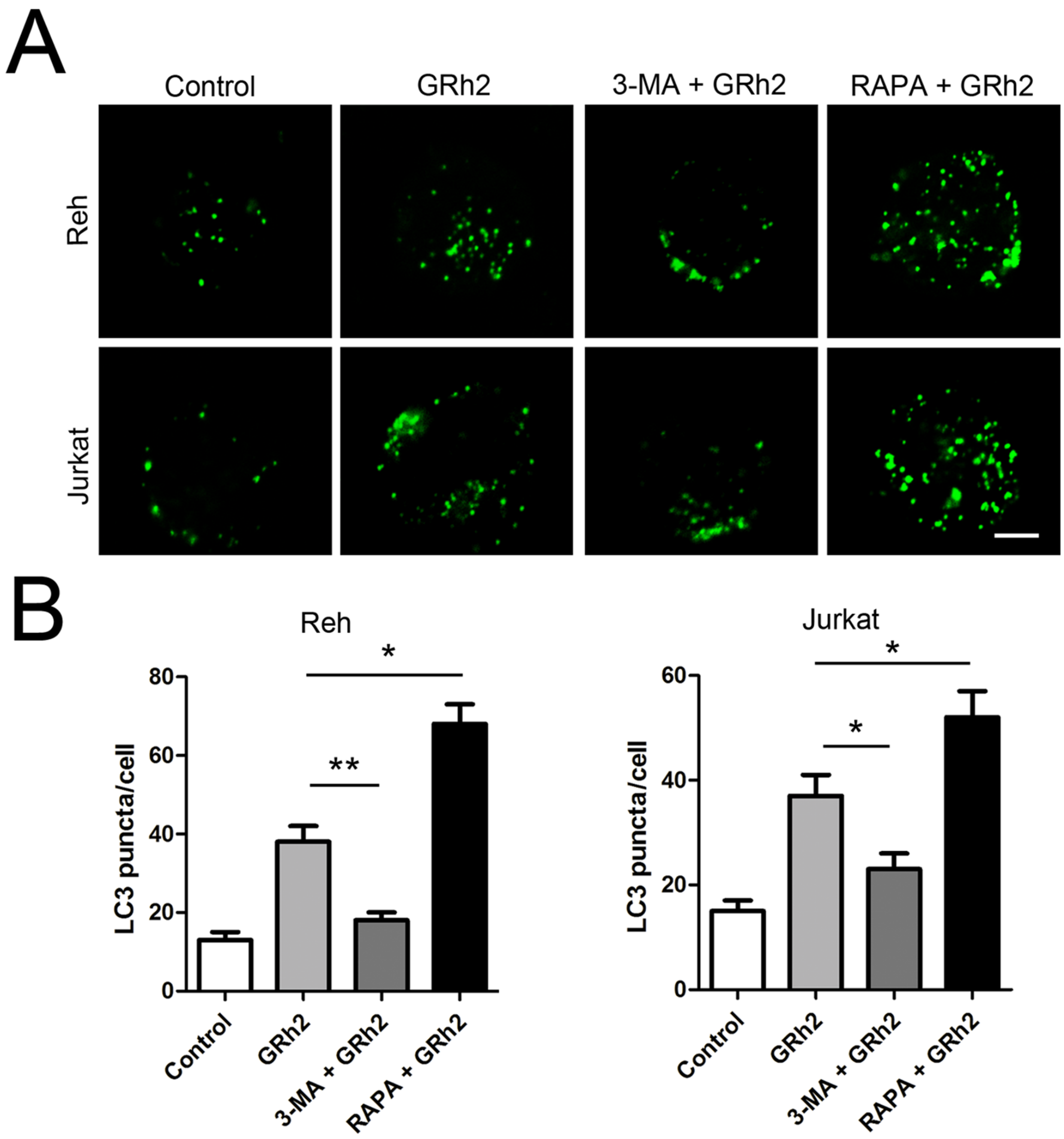

Supplementary Figure S4: The involvement of autophagy was confirmed using LC3 puncta assay. Cells were treated with 40 µM 20(S)-GRh2 in the presence or absence of 3-MA or RAPA for 24 h. **A.** In Reh and Jurkat cells, the fluorescence of GFP-LC3 was examined by laser scanning confocal microscope (Bar=5 µM). **B.** GFP-LC3 puncta in each cell was determined and bar graphs are representative of 100 cells. Data are presented as mean ± SEM (n = 3) for each group. * $p < 0.05$, ** $p < 0.01$.

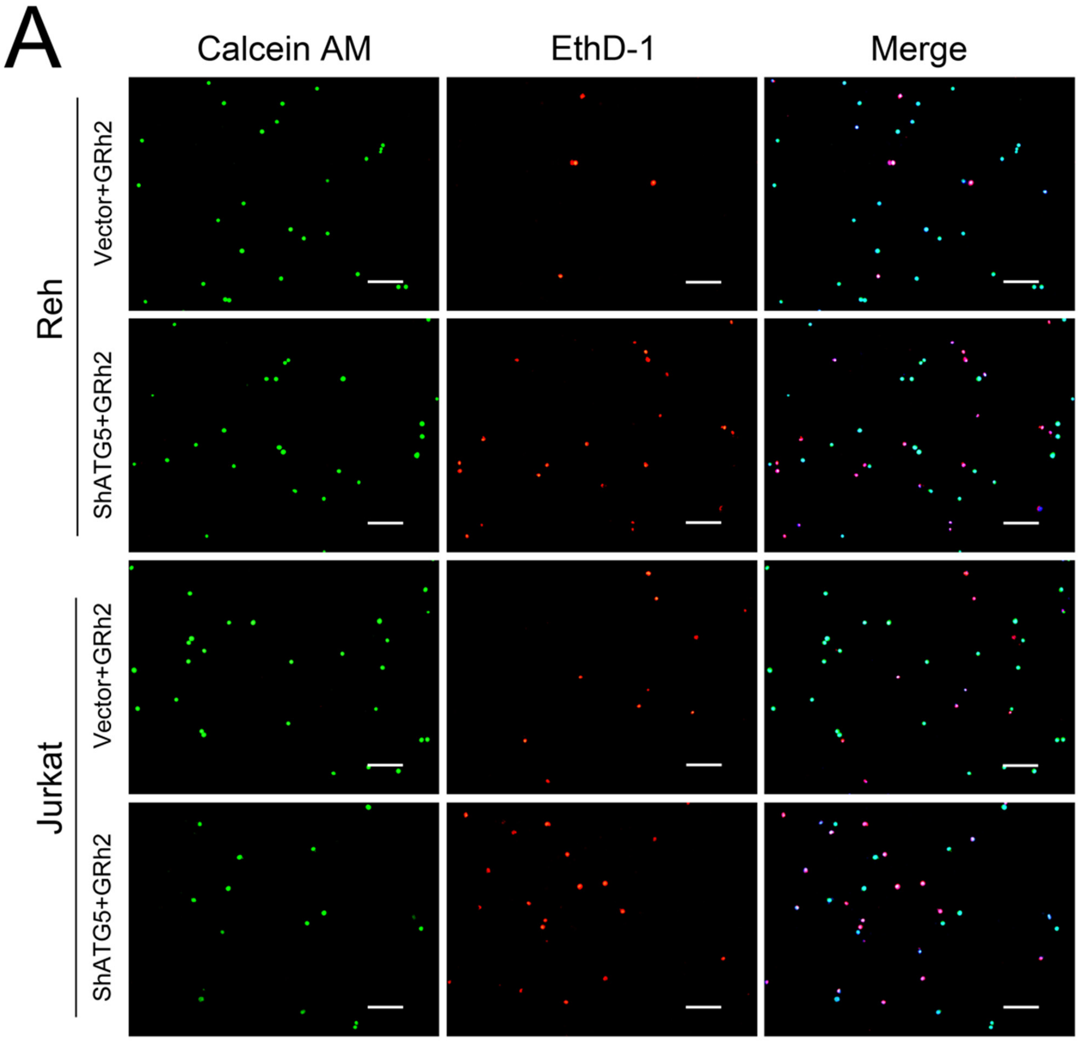

Supplementary Figure S5: 20(S)-GRh2-induced cytotoxicity was determined by the LIVE/DEAD® viability/ cytotoxicity assay. Cells transfected with vector or ShATG5 plasmid were exposed to 40 μM 20(S)-GRh2 for 24 h. Cells were stained with calcein AM, EthD-1 and Hoechst 33342. The percentage of dead cells with EthD-1 probe was determined in high-power fields (Bar=50 μM) of each sample.

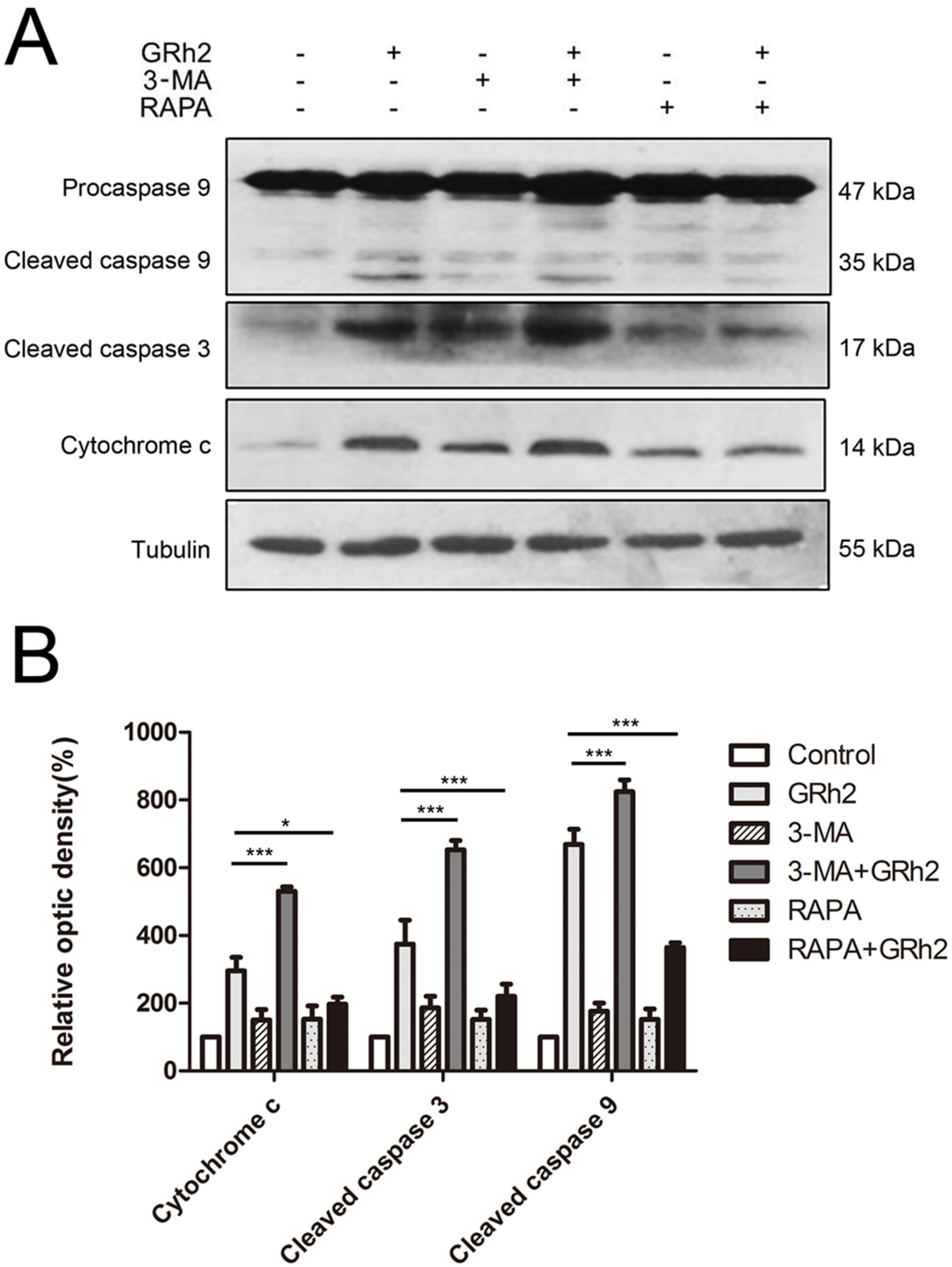

Supplementary Figure S6: Expression of mitochondria-associated pro-apoptotic proteins with regulation of autophagy. Reh cells were treated with 40 μM GRh2 in the presence or absence of 3-MA or RAPA for 24 h. **A.** The expression of cytochrome c and cleavage of caspase-9, -3 were detected by western blot analysis. Antibody against Tubulin served as a loading control. **B.** The corresponding histograms were quantified by Image J. Data are reported as mean ± SEM (n=3) for each group. * $p < 0.05$, *** $p < 0.001$.

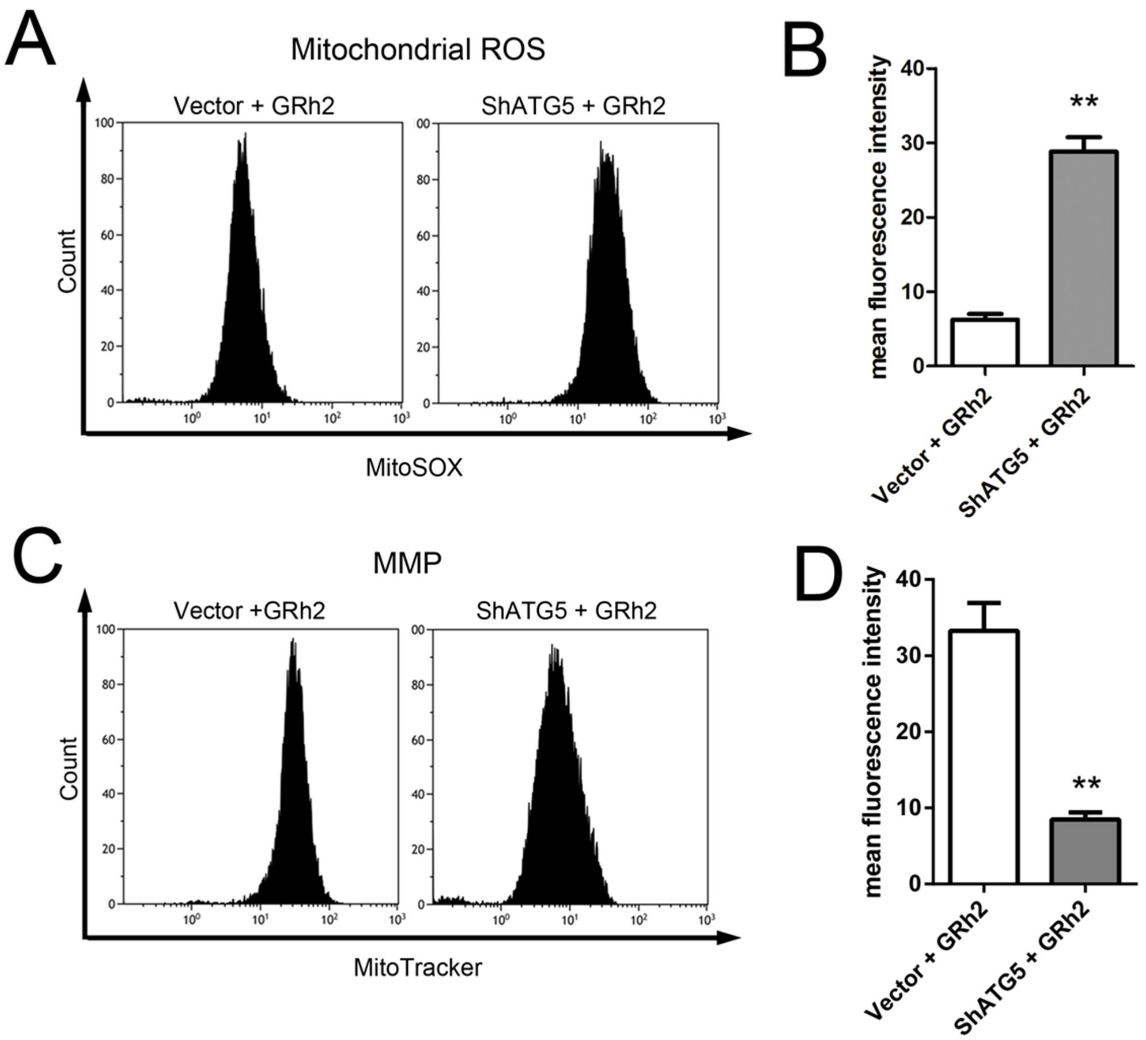

Supplementary Figure S7: ATG5 knockdown exacerbates 20(S)-GRh2-induced cell death via mitochondrial ROS and mitochondrial damage. Reh cells were treated with GRh2 + shATG5 or GRh2 + vector for 24 h. **A.** The MitoSOX™ Red fluorescence intensity was detected by flow cytometry. **B.** The corresponding histograms were quantified by Image J. All data are represented as mean ± SEM (n = 3) for each group. ** $p < 0.01$. **C.** Cells were loaded with red-fluorescent MitoTracker probe. The fluorescence intensity was detected by flow cytometry. **D.** The mean fluorescence intensity reflects MMP levels. Data are represented as mean ± SEM (n=3) for each group. ** $p < 0.01$.
